# Response‐adapted consolidation and salvage strategies in secondary central nervous system lymphoma: Insights from a multicentre cohort

**DOI:** 10.1111/bjh.70633

**Published:** 2026-06-25

**Authors:** Farina Eigendorff, Fabian Görke, Vanja Zeremski, Sophia Wollnitza, Evgenii Shumilov, Georg Lenz, Ellen Obstfelder, Inken Hilgendorf, Vladan Vučinić, Andreas Hochhaus, Ulf Schnetzke

**Affiliations:** ^1^ Klinik für Innere Medizin II, Abteilung für Hämatologie und Internistische Onkologie, Comprehensive Cancer Center Central Germany—Campus Jena Universitätsklinikum Jena Jena Germany; ^2^ Klinik und Poliklinik für Hämatologie, Zelltherapie, Hämostaseologie und Infektiologie, Comprehensive Cancer Center Central Germany—Campus Leipzig Universitätsklinikum Leipzig Leipzig Germany; ^3^ Klinik für Hämatologie, Onkologie und Zelltherapie Universitätsklinikum Magdeburg Magdeburg Germany; ^4^ Medizinische Klinik A, Hämatologie, Hämostaseologie, Onkologie und Pneumologie Universitätsklinikum Münster Münster Germany

**Keywords:** CAR T therapy, high‐dose chemotherapy, prognostic factors, secondary CNS lymphoma

## Abstract

Secondary central nervous system lymphoma (SCNSL) remains a rare but challenging manifestation of aggressive lymphomas, with limited prospective data to guide treatment decisions in the modern therapeutic era. We conducted a multicentre retrospective cohort study of 105 consecutively treated adult patients diagnosed with SCNSL between 2016 and 2024 across four German academic centres, capturing detailed treatment sequencing, response dynamics and outcome data. Median progression‐free survival (PFS) was 7.62 months (95% CI 5.34–9.90), and median overall survival (OS) was 16.62 months (95% CI 5.91–27.33). Consolidation with high‐dose chemotherapy and autologous haematopoietic stem cell transplantation (HDT‐AHSCT) was associated with significantly improved OS and PFS in univariate and multivariate analyses. Achieving complete remission prior to HDT‐AHSCT conferred with superior outcomes. In a small subset of patients with relapsed/refractory (rr) SCNSL, CD19‐directed chimeric antigen receptor T‐cell (CAR T) therapy showed favourable outcomes compared with non‐transplant salvage therapy in exploratory univariate analysis (OS HR 0.28, 95% CI 0.08–0.94, *p* = 0.039; PFS HR 0.37, 95% CI 0.16–0.88, *p* = 0.025). In this contemporary multicentre real‐world cohort, early response and consolidative HDT‐AHSCT emerged as key determinants of durable disease control, while CAR T therapy may represent a salvage strategy for selected patients. These findings provide clinically relevant benchmarks to inform risk‐adapted treatment sequencing in SCNSL.

## INTRODUCTION

Secondary involvement of the central nervous system by aggressive lymphomas is uncommon but clinically formidable, associated with poor survival.^1^, ^2^, ^3^, ^4^ Central nervous system (CNS) dissemination occurs in approximately 4%–6% of patients with diffuse large B‐cell lymphoma (DLBCL), the most common lymphoma associated with secondary central nervous system lymphoma (SCNSL).^4^

Optimal management remains controversial, as randomized trials are lacking and patients with CNS involvement have been largely excluded from prospective trials. Treatment paradigms are therefore extrapolated from primary CNS lymphoma (PCNSL) and retrospective SCNSL series.^1^, ^3^, ^5^, ^6^, ^7^, ^8^, ^9^, ^10^ In PCNSL, high‐dose chemotherapy with autologous haematopoietic stem cell transplantation (HDT‐AHSCT) improves long‐term disease control and this approach is frequently applied to eligible patients with SCNSL.^11^, ^12^, ^13^ The phase II, IELSG42/MARIETTA trial provided the strongest prospective evidence for intensive treatment in SCNSL.^4^ A 7‐year follow‐up analysis demonstrated a progression‐free survival (PFS) rate of 33% for all patients, 52% for de novo SCNSL and 64% for HDT‐AHSCT recipients.^14^ The 7‐year overall survival (OS) rates were 33%, 52% and 64% for the whole cohort, de novo SCNSL and HDT‐AHSCT recipients respectively. Recently, Alderuccio et al. reported the largest cohort to date evaluating treatment‐related outcomes and patterns of relapse in secondary CNS involvement by DLBCL underscoring the importance of intensive methotrexate‐containing induction therapy and consolidative HDT‐AHSCT for selected patients.^1^

Anti‐cluster of differentiation 19 (CD19) chimeric antigen receptor T‐cell (CAR T) therapy has improved treatment of relapsed/refractory (rr) DLBCL. Although patients with secondary CNS involvement were largely excluded from pivotal CAR T trials, accumulating real‐world data suggest that CAR T cells can traffic to and act within the CNS without excessive neurotoxicity.^15^, ^16^

We therefore performed a multicentre retrospective study of SCNSL patients across four German tertiary academic care centres. We analysed real‐world treatment strategies and outcomes, including consolidative HDT‐AHSCT, CAR T therapy compared with conventional salvage regimens and prognostic factors. Our data show that intensive approaches including HDT‐AHSCT are associated with prolonged survival, particularly in patients achieving complete remission (CR) before HDT‐AHSCT, and suggest a potential role for CAR T therapy in selected pretreated patients. This large multicentre cohort provides real‐world evidence on contemporary treatment strategies.

## MATERIALS AND METHODS

### Patient cohort and informed consent

This cohort included 105 consecutive adult patients treated for SCNSL from January 2019 to December 2024 at four German tertiary academic hospitals. All patients were ≥18 years and diagnosed with SCNSL arising from histologically confirmed large B‐cell lymphoma (LBCL), defined as first CNS involvement. CNS involvement encompassed parenchymal, meningeal, intraspinal/intradural, ocular or combined manifestations and was confirmed histologically via biopsy, by cerebrospinal fluid analysis or radiologically by computed tomography (CT) or magnetic resonance imaging (MRI). SCNSL was classified as synchronous (CNS involvement at initial systemic lymphoma diagnosis) or metachronous (progression/relapse after systemic lymphoma diagnosis, either isolated or concomitant with systemic manifestation).

Response assessment for initial systemic lymphoma was performed using either CT or positron emission tomography (PET)/CT, depending on local standard of care. Patients treated within clinical trials were excluded.

All patients or their legal representatives provided informed consent. This study was approved by the local Ethics Committee (Reg No. 2025‐3038‐BO‐D) and performed in accordance with the Declaration of Helsinki.

### Data collection and variables

Clinical data were collected from institutional patient records. Comprehensive treatment data encompassed all therapy lines and protocols administered for initial therapy (for patients without SCNSL at initial diagnosis) and for SCNSL therapy, including corresponding response assessments. Second‐line and subsequent therapies for SCNSL were analysed exclusively for patients with ongoing CNS involvement; patients experiencing isolated systemic relapse following SCNSL‐directed therapy were censored. CAR‐T and ibrutinib therapy were only analysed if administered for CNS disease.

Time to SCNSL was defined as the interval between initial lymphoma diagnosis and SCNSL manifestation. Time to first progression (TTP1) was calculated from initial lymphoma diagnosis or SCNSL diagnosis until first progression/relapse. Patients without progression/relapse were censored at death or last follow‐up, up to data cut‐off. Lymphoma therapy prior to CNS manifestation is shown in Tables S1 and S2. For SCNSL‐directed therapy, only relapses with CNS involvement were included. TTP2 was calculated from first relapse until second progression/relapse, with patients without second event censored at death or last follow‐up. TTP3 and TTP4 were defined analogously. PFS was defined from SCNSL manifestation to the date of first documented relapse, progressive disease or death. OS was defined from SCNSL manifestation to death or last follow‐up. Analyses of PFS and OS used data up to cut‐off date of 01 May 2025.

### Statistics

Patient characteristics were summarized using descriptive statistics, reporting frequencies and percentages for categorical variables and medians for continuous variables. Probabilities of OS and PFS were estimated using Kaplan–Meier plots. Patients with missing OS data were excluded. Differences between survival curves were assessed with the log‐rank test. For parameters showing significant differences in the log‐rank test (*p* ≤ 0.05), univariate Cox proportional hazards regression was performed. A multivariable Cox proportional hazards regression model was conducted to identify independent prognostic factors, including age (per 10‐year increment), Eastern Cooperative Oncology Group (ECOG) performance status (PS) at SCNSL diagnosis, Ann Arbor stage at initial diagnosis, extra‐nodal manifestation and testicular/kidney manifestation at initial diagnosis. HDT‐AHSCT was entered into the multivariable Cox regression model as a fixed binary covariate (ever vs. never exposed), acknowledging potential immortal bias, as patients who receive HDT‐AHSCT are required to survive event‐free until the time of transplantation. Cases with missing data in the multivariable Cox analyses were excluded (complete case analysis). To evaluate the impact of high‐dose methotrexate (HD‐MTX) and intrathecal chemotherapy and SCNSL localization on survival, survival curves were compared using log‐rank tests. All tests were two sided with a level of significance of 0.05. Analyses were performed using SPSS 30.0 (SPSS Inc., Chicago, IL, USA).

## RESULTS

### Patients' characteristics

A total of 105 patients with SCNSL were included, with baseline characteristics summarized in Table 1. Median age at CNS involvement was 65 years (range, 19–84), and 51 patients (48.6%) were female. At initial lymphoma diagnosis, 78 patients (76.5%) had advanced‐stage disease (Ann Arbor stage III–IV) and among patients with available International Prognostic Index (IPI), 61 (73.5%) had an intermediate‐high/high IPI score (IPI ≥3). Extra‐nodal disease outside the CNS was present in 86 patients (81.9%), bulky disease in 23 (21.9%) and testicular involvement in 10 (8.3%). CNS‐IPI risk was low in 7 (8.6%), intermediate in 39 (48.1%) and high in 35 patients (43.2%). The median ECOG PS at initial lymphoma diagnosis was 1 (range 0–4), with 71.8% ECOG 0–1 and 28.2% ECOG ≥2.

**TABLE 1:** Patients' characteristics.

Age at SCNSL diagnosis (years, median [range])	—	65 (19–84)
Sex, *n* (%)	Female	51 (48.6)
	Male	54 (51.4)
Lymphoma entity (CNS), *n* (%)	LBCL	105 (100)
Transformed lymphoma, *n* (%)	Indolent to aggressive	15 (14.3)
Ann Arbor stage at initial diagnosis, *n* (%)	I	11 (10.8)
	II	13 (12.7)
	III	7 (6.9)
	IV	71 (69.6)
	n.d.	3
IPI at initial lymphoma diagnosis, *n* (%)	0–2 (low/low–intermediate)	22 (26.5)
	3–5 (high–intermediate/high)	61 (73.5)
	n.d.	22
CNS‐IPI at initial lymphoma diagnosis, *n* (%)	0–1 (low)	7 (8.6)
	2–3 (intermediate)	39 (48.1)
	4–6 (high)	35 (43.2)
	n.d.	24
ECOG PS at initial lymphoma diagnosis, median (range)	—	1 (0–4)
ECOG PS at initial lymphoma diagnosis, *n* (%)	0	18 (23.1)
	1	38 (48.7)
	2	17 (21.8)
	3	4 (5.1)
	4	1 (1.3)
	n.d.	27
Patients with ENM at initial lymphoma diagnosis (except CNS), *n* (%)	—	86 (81.9)
ENM localization at initial lymphoma diagnosis, *n* (%)	Bone marrow	24 (19.8)
	Gastrointestinal/peritoneal	21 (17.4)
	Pulmonal/pleural	12 (9.9)
	Head/neck	11 (9.1)
	Kidney/adrenal	11 (9.1)
	Bone	10 (8.3)
	Testes	10 (8.3)
	Muscle/soft tissue	6 (5.0)
	Liver	6 (5.0)
	Cardiac	5 (4.1)
	Skin	3 (2.5)
	Mamma	2 (1.7)
Bulk manifestation at initial lymphoma diagnosis, *n* (%)	—	23 (21.9)
Testicle manifestation at any time, *n* (%)	—	10 (9.5)
Patients with SCNSL manifestation at initial diagnosis, *n* (%)	—	22 (21.0)
Patients without SCNSL manifestation at initial diagnosis, *n* (%)	—	83 (79.0)

Abbreviations: CNS IPI, Central Nervous System International Prognostic Index; CNS, central nervous system; ECOG PS, Eastern Cooperative Oncology Group Performance Status; ENM, extra‐nodal manifestation; FL, follicular lymphoma; IPI, International Prognostic Index; LBCL, large B‐cell lymphoma; n.d., no data; SCNSL, secondary central nervous system lymphoma.

### SCNSL characteristics

Of 105 patients, 22 (21.0%) presented with CNS involvement at initial lymphoma diagnosis (‘synchronous’ SCNSL), while 83 (79.0%) developed CNS relapse or progression after remission of initial systemic lymphoma (‘metachronous’ SCNSL) (Table 1). Among metachronous cases, median latency to CNS manifestation was 8.4 months (range, 0.1–140 months), with 80.0% occurring within 24 months (Table 2). At SCNSL diagnosis, median ECOG PS had declined to 2 (range, 0–4), and 57 patients (55.3%) had ECOG ≥2. CNS involvement was most commonly parenchymal (*n* = 60, 57.1%), followed by leptomeningeal disease (*n* = 32, 30.5%). Concurrent systemic lymphoma was present in 33 patients (31.4%), while 72 patients (68.6%) had an isolated CNS relapse. Prior to CNS involvement, 71 patients (67.6%) had received one line of systemic therapy and 12 (11.5%) had received ≥2 lines. CNS prophylaxis had been administered to 16/83 patients with metachronous SCNSL (19.3%), most commonly intravenous HD‐MTX (*n* = 12) or intrathecal chemotherapy (*n* = 4).

**TABLE 2:** SCNSL data.

Latency from initial diagnosis to SCNSL manifestation (months), median (range)	—	8.4 (0.1–140)
Time point of SCNSL, *n* (%)	Early onset (≤24 months after initial diagnosis)	84 (80.0)
	Late onset (>24 months after initial diagnosis)	21 (20.0)
SCNSL localization, *n* (%)	Parenchymal	60 (57.1)
	Meningeal	32 (30.5)
	Ocular	1 (1.0)
	Spinal cord	1 (1.0)
	Combined	11 (10.4)
Concomitant systemic relapse/manifestation, *n* (%)	Yes	33 (31.4)
	No	72 (68.6)
ECOG PS at SCNSL diagnosis, median (range)	—	2 (0–4)
ECOG PS at SCNSL diagnosis, *n* (%)	0	5 (4.9)
	1	41 (39.8)
	2	32 (31.1)
	3	16 (15.5)
	4	9 (8.7)
	Unknown	2
Lines of therapy before SCNSL manifestation^a^ (range)	—	1 (0–4)
Lines of therapy before SCNSL manifestation, *n* (%)	0	22 (21.0)
	1	71 (67.6)
	2	5 (4.8)
	3	5 (4.8)
	4	2 (1.9)
CNS prophylaxis at initial treatment, *n* (%)	Total	16 (19.3)
	(R‐) HD‐MTX (+ifosfamide)	12 (14.5)
	Intrathecal MTX or triple therapy	4 (4.8)
Reason of death	Progression	30 (58.8)
	Infectious complications	13 (25.5)
	Other^b^	8 (15.7)

Abbreviations: (R‐) HD‐MTX, (rituximab‐) high‐dose methotrexate; CNS, central nervous system; ECOG PS, Eastern Cooperative Oncology Group Performance Status; SCNSL, secondary central nervous system lymphoma.

^a^ Only patients without SCNSL manifestation at initial diagnosis.

^b^ Heart failure (*n* = 4), acute liver failure (*n* = 1), secondary malignancy (*n* = 1), unknown (*n* = 2).

### Treatment of initial peripheral lymphoma diagnosis

First‐line treatment of the 83 patients with metachronous SCNSL is shown in Table S1. Most patients received R‐CHOP/R‐CHOEP immunochemotherapy (*n* = 75, 90.3%), with 12.0% also receiving consolidative radiotherapy. Best responses were CR in 53 patients (67.1%) and partial remission (PR) in 16 (20.3%). Subsequent treatment before CNS involvement is summarized in Table S2.

### Treatment and response of SCNSL

Overall, 102/105 patients (97.1%) received at least one line of CNS‐directed therapy. Most received one (*n* = 56, 53.3%) or two lines (*n* = 34, 32.4%) (Table 3). Common first‐line regimens were MATRix (HD‐MTX, cytarabine, thiotepa, rituximab) (34.3%), R‐MP (rituximab, high‐dose MTX ± procarbazine) (29.5%) and R‐CHOP with CNS‐directed additions (12.4%). Intrathecal (IT)‐chemotherapy was added in 17.1%, radiotherapy in 11.4% and consolidation HDT‐AHSCT (mainly thiotepa‐based conditioning regimens) following first‐line treatment in 25.7% (*n* = 27). CAR T therapy was used as part of initial SCNSL treatment in two patients. In both cases, patients experienced early relapse with concomitant systemic involvement and were considered eligible for CAR T therapy. The overall response rate (ORR) to first‐line therapy was 68.6% (CR: 45.7%, PR: 22.9%), while 14.3% died or progressed before response assessment.

**TABLE 3:** Therapy for SCNSL manifestation.

Lines of therapy for SCNSL incl. BSC, *n* (%)	1	56 (53.3)
	2	34 (32.4)
	3	11 (10.5)
	4	2 (1.9)
	≥5	2 (1.9)
First‐line therapy, *n* (%)	MATRix	36 (34.3)
	RM(P)	31 (29.5)
	(R)‐CHOP	13 (12.4)
	Other^a^	25 (23.8)
Additional therapy to first‐line therapy, *n* (%)	Add. HDT‐AHSCT	27 (25.7)
	Add. intrathecal therapy	18 (17.1)
	Add. radiation	12 (11.4)
	Add. R‐MTX	8 (7.6)
	Add. CAR T	2 (1.9)
	Other^b^	2 (1.9)
	Total	69 (65.7)
Best response to first‐line therapy, *n* (%)	CR	48 (45.7)
	PR	24 (22.9)
	SD	6 (5.7)
	PD	12 (11.4)
	Not assessed	15 (14.3)
TTP1 (months), median (95% CI)	—	8.61 (6.04–11.17)
Lymphoma manifestation after first‐line therapy, *n* (%)	SCNSL manifestation	52 (74.3)
	SCNSL only	32 (45.7)
	SCNSL with concomitant systemic manifestation	17 (24.3)
	Systemic only	13 (18.6)
	Not assessed	5 (7.1)
Second‐line therapy, *n* (%)	Radiation only	13 (25.0)
	BSC	10 (19.2)
	MATRix	4 (7.7)
	(R)‐DeVIC	3 (5.8)
	Ibrutinib	3 (5.8)
	RM(P)	3 (5.8)
	CAR T	2 (3.8)
	Other^c^	14 (26.9)
Additional therapy to second‐line therapy, *n* (%)	Add. intrathecal therapy	8 (15.4)
	Add. radiation	5 (9.6)
	Add. CAR T	5 (9.6)
	Add. HDT‐AHSCT	3 (5.8)
	Add. allogenic PBHSCT	1 (1.9)
	Total	20 (38.5)
Best response to second‐line therapy, *n* (%)	CR	11 (20.4)
	PR	10 (18.5)
	SD	5 (9.3)
	PD	5 (9.3)
	Not assessed	23 (42.6)
TTP2 (months), median (95% CI)	—	4.47 (0.86–8.07)
Lymphoma manifestation after second‐line therapy, *n* (%)	SCNSL manifestation	21 (80.8)
	SCNSL only	19 (73.1)
	SCNSL with concomitant systemic manifestation	2 (7.7)
	Systemic only	5 (19.2)
HDT‐AHSCT for SCNSL, *n* (%)	Yes	31 (29.5)
	No	74 (70.5)
HDT‐regime, *n* (%)	BCNU/thiotepa	20 (64.5)
	TBC	5 (16.1)
	R‐TEAM	3 (9.7)
	R‐BEAM	2 (6.5)
	Unknown	1 (3.2)
Remission prior HDT‐AHSCT, *n* (%)	CR	16 (51.6)
	PR	14 (45.1)
	SD	0 (0)
	PD	0 (0)
	Not assessed	1 (3.2)
CAR T for SCNSL, *n* (%)	Yes	9 (8.6)
	No	96 (91.4)

Abbreviations: (R)‐DeVIC, (rituximab), dexamethasone, etoposide, ifosfamide, carboplatin; AraC, high‐dose cytarabine; BCNU, carmustine; BSC, best supportive care; CAR T, chimeric antigen receptor T‐cell; CR, complete remission; HDT‐AHSCT, high‐dose chemotherapy with autologous haematopoietic stem cell transplantation; IFO, ifosfamide; MATRix, high‐dose methotrexate, cytarabine, thiotepa, rituximab; PBHSCT, peripheral blood haematopoietic stem cell transplantation; PR, partial remission; R‐BEAM, rituximab, carmustine, etoposide, cytarabine, melphalan; R‐DHAOx, rituximab, dexamethasone, high‐dose cytarabine, oxaliplatin; R‐DHAP, rituximab, dexamethasone, high‐dose cytarabin, cisplatin; RM(P), rituximab, high‐dose methotrexate, (procarbazine); R‐TEAM, rituximab, thiotepa, etoposide, cytarabine, melphalan; SCNSL, secondary central nervous system lymphoma; TBC, thiotepa, busulfan, cyclophosphamide; TT, thiotepa; TTP1, time to first progression.

^a^ R‐DHAP (*n* = 3), BSC (*n* = 3), rituximab/MTX/ifosfamide/AraC/dexamethasone + AraC/thiotepa/dexamethasone + AraC intrathecal (Berliner protocol) (*n* = 2), (R)‐AraC/dexamethasone (*n* = 3), radiation only (*n* = 2), R‐MTX/IFO (*n* = 2), R‐MTX/AraC/TT (*n* = 7), R‐(Pola)‐bendamustine (*n* = 1), R‐MTX/IFO/AraC/dexametasone (*n* = 1), MATRix/R‐AraC/TT (Freiburger protocol) (*n* = 1).

^b^ Immunochemotherapy (*n* = 1), intravitreal rituximab (*n* = 1).

^c^ R/AraC/TT (*n* = 4), R‐Pola‐Benda (*n* = 1), tafasitamab/lenalidomide (*n* = 2), R‐CHOP (*n* = 1), R‐DHAOx (*n* = 1), MTX/AraC/TT (*n* = 1), obinutuzumab/glofitamab (*n* = 1), epcoritamab (*n* = 1), unknown (*n* = 2).

Among 70 patients with progression or no response, 52 had CNS relapse, and 42 received second‐line therapy and 10 best supportive care (BSC). Treatments were heterogeneous and are illustrated in Table 3. CAR T therapy was administered in second line either as primary treatment in two patients or as consolidative therapy in five patients. At CAR T indication, four patients had isolated CNS disease and five had concomitant CNS/systemic involvement. Other consolidative approaches after second‐line therapy included HDT‐AHSCT, radiotherapy and allogeneic HSCT (allo‐HSCT) in 19 patients. Second‐line ORR was 38.9% (CR: 20.4%, PR: 18.5%); 42.6% were not evaluable. Median PFS after second‐line therapy was 4.47 months (95% confidence interval (CI) 0.86–8.07), with only 2/28 evaluable patients progression‐free at 1 year.

Beyond second line, 21 patients (80.8%) had recurrent CNS involvement (Table 3). Eight received BSC, and median PFS after third line was 2.79 months (95% CI 0.97–4.62). One patient achieved CR after fourth‐line allo‐HSCT, subsequently relapsed and achieved durable CR with epcoritamab (Table S3).

### Survival outcomes

**FIGURE 1:**
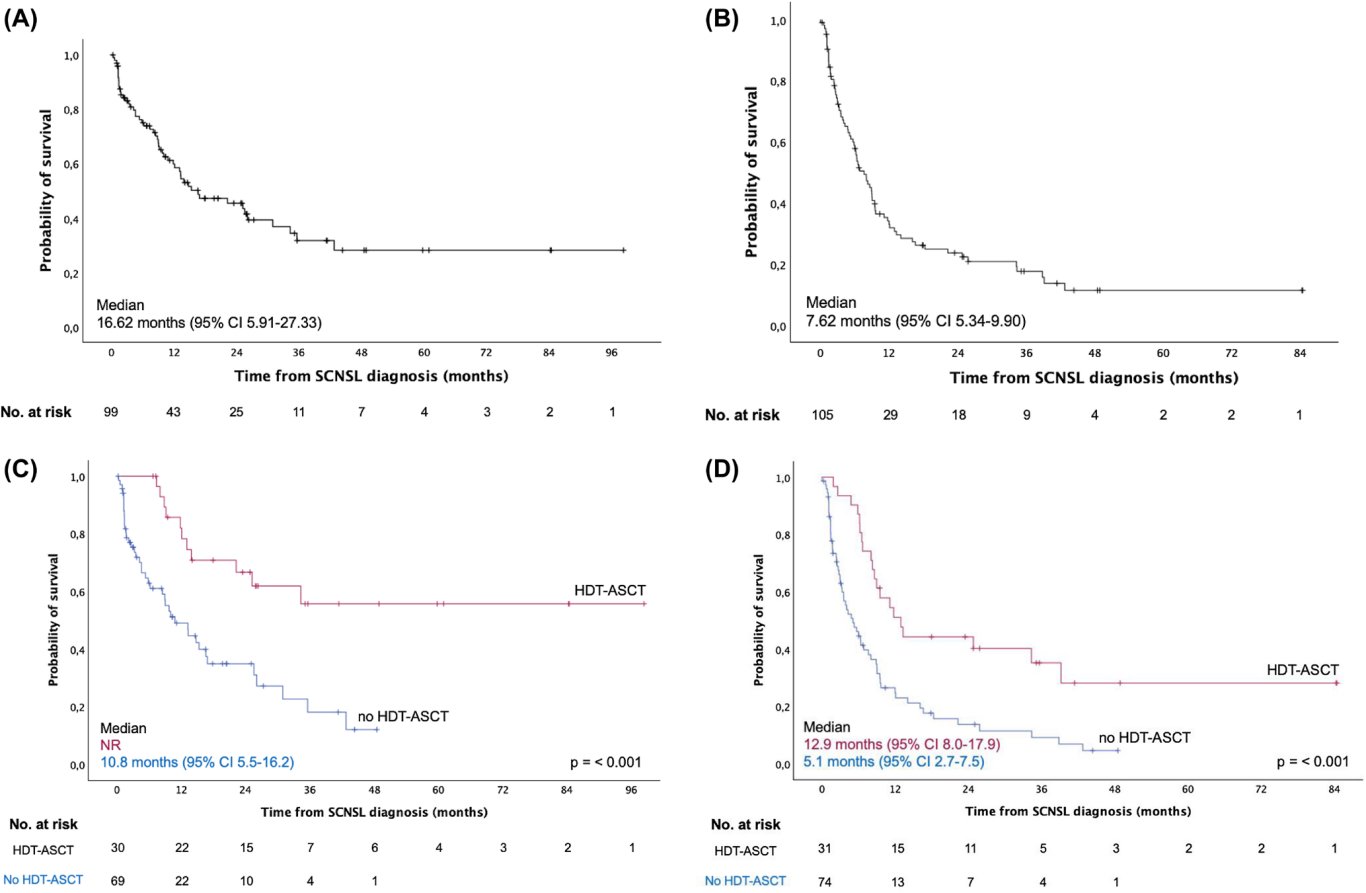
(A) Overall survival (OS) from secondary central nervous system lymphoma (SCNSL) diagnosis. 1‐year OS 58.6%; 2‐year OS 44.0%; Median follow‐up (months) 25.9; data cut‐off 01.05.2025, 47 patients alive and censored. (B) Progression‐free survival (PFS) from SCNSL diagnosis. 1‐year PFS 31.9%; 2‐year PFS 23.0%; Median follow up (months) 35.7; data cut‐off 01.05.2025, 25 patients progression‐free and censored. (C) OS from SCNSL diagnosis. High‐dose chemotherapy with autologous haematopoietic stem cell transplantation (HDT‐AHSCT) versus no HDT‐AHSCT. (D) PFS from SCNSL diagnosis. HDT‐AHSCT versus no HDT‐AHSCT.

At the data cut‐off (1 May 2025), 47/105 patients (52.1%) were alive and 52 (47.9%) had died, mainly from disease progression (*n* = 30, 58.8%) or infectious complications (*n* = 13, 25.5%) (Table 2). OS data were unavailable for six patients. Median OS from SCNSL diagnosis for the entire cohort was 16.62 months (95% CI, 5.91–27.33 months), with 1‐ and 2‐year OS rates of 58.6% and 44.0% respectively (Figure 1A). Median PFS was 7.62 months (95% CI, 5.34–9.90 months), with 1‐ and 2‐year PFS rates of 31.9% and 23.0% (Figure 1B). Median follow‐up for OS and PFS was 25.9 and 35.7 months respectively.

In univariate analysis, ECOG 0–1 at SCNSL diagnosis was associated with superior OS and PFS compared with ECOG ≥2 (median OS 31.0 vs. 13.3 months, *p* = 0.025; median PFS 11.8 vs. 5.9 months, *p* < 0.001; Table 4). Patients with ≥2 prior systemic therapy lines had inferior OS and PFS compared to those with synchronous SCNSL or first relapse (median OS 4.6 vs. 34.3 months, *p* = 0.012; median PFS: 3.0 vs. 6.5 months, *p* = 0.020). Concomitant systemic relapse was associated with inferior OS (median OS 10.8 vs. 31.0 months; *p* = 0.041).

**TABLE 4:** Association of OS and PFS with clinical parameters (log‐rank test).

Parameter	Versus	Log rank OS (*n* = 99)	Log rank PFS (*n* = 105)
Sex	Female vs. male	0.118	0.263
Ann Arbor at initial lymphoma diagnosis (*n* = 102)	1–2 vs. 3–4	0.970	0.598
IPI at initial lymphoma diagnosis (*n* = 83)	Low/low–intermediate vs. high–intermediate/high	0.388	0.182
CNS IPI at initial lymphoma diagnosis (*n* = 81)	Low vs. intermediate/high	0.358	0.298
ENM at initial lymphoma diagnosis	Yes vs. no	0.800	0.341
Bulk manifestation at initial lymphoma diagnosis	Yes vs. no	0.875	0.265
Testicle involvement at any time	Yes vs. no	0.286	0.560
Kidney/adrenal gland involvement at initial lymphoma diagnosis	Yes vs. no	0.102	0.208
SCNSL at initial lymphoma diagnosis	Synchronous vs. metachronous	0.826	0.312
Time point SCNSL	Early (≤24 months) vs. late onset (>24 months)	0.824	0.086
ECOG at SCNSL manifestation (*n* = 104)	0–1 vs. ≥2	**0.025**	**<0.001**
SCNSL localization	Parenchymal vs. meningeal vs. ocular vs. spinal vs. combined	0.075	**0.036**
Concomitant systemic relapse	Yes vs. no	**0.041**	0.145
Lines of therapy at initial lymphoma diagnosis^a^ (*n* = 83)	0–1 vs. ≥2	**0.012**	**0.020**
CNS prophylaxis^a^ (*n* = 83)	Yes vs. no	0.969	0.936
Best response to first‐line SCNSL (*n* = 90)	CR/PR vs. SD/PD	**<0.001**	**<0.001**
HDT‐AHSCT for SCNSL	Yes vs. no	**<0.001**	**<0.001**
Remission prior HDT‐AHSCT^b^ (*n* = 30)	CR vs. PR	**0.029**	**0.003**
CAR T from second‐line SCNSL onwards^c^ (*n* = 56)	Yes vs. no	**0.034**	**0.023**
Ibrutinib from second‐line SCNSL onwards^c^ (*n* = 56)	Yes vs. no	0.691	0.556

*Note*: Bold values indicates *p* ≤ 0.05.

Abbreviations: CAR T, chimeric antigen receptor T‐cell; CNS, central nervous system; CR, complete remission; ECOG PS, Eastern Cooperative Oncology Group Performance Status; ENM, extra‐nodal manifestation; HDT‐AHSCT, high‐dose chemotherapy with autologous haematopoietic stem cell transplantation; IPI, International Prognostic Index; OS, overall survival; PFS, progression‐free survival; PR, partial remission; SCNSL, secondary central nervous system lymphoma.

^a^ Only patients without SCNSL manifestation at initial diagnosis.

^b^ Only patients with HDT‐AHSCT, all patients with consolidative HD‐AHSCT achieved either PR or CR following induction chemotherapy.

^c^ Only patients with progression/relapse after first‐line SCNSL therapy and concomitant CNS involvement at progression/relapse.

SCNSL manifestation pattern was associated with PFS in exploratory analysis. Parenchymal involvement was associated with longer PFS than meningeal or combined CNS involvement (both *p* = 0.019) (Tables S7 and S8; Figure S1). Baseline factors including Ann Arbor stage, IPI, CNS‐IPI, extra‐nodal sites, bulky disease, immune‐privileged site involvement or CNS prophylaxis were not significantly associated with survival.

Response to first‐line SCNSL therapy was strongly prognostic. Patients achieving CR/PR had superior OS compared with stable disease/progressive disease (SD/PD) (26.1 vs. 4.6 months, *p* < 0.001), with 2‐year OS rates of 56.7% vs. 20.8%. Cox regression confirmed this association for OS (hazard ratio (HR) 0.24, 95% CI 0.12–0.48, *p* < 0.001) and PFS (HR 0.08, 95% CI 0.04–0.16, *p* < 0.001) (Table 5).

**TABLE 5:** Association of OS and PFS with clinical parameters (univariate Cox regression analysis).

Parameter	Versus	Univariate cox regression OS (HR [95% CI], *p*) (*n* = 99)	Univariate cox regression PFS (HR [95% CI], *p*) (*n* = 105)
ECOG at SCNSL manifestation (*n* = 104)	0–1 vs. ≥2	0.53 (0.30–0.93), ***p* = 0.028**	0.45 (0.28–0.71), ***p* < 0.001**
Concomitant systemic relapse	Yes vs. no	1.77 (1.01–3.08), ***p* = 0.044**	1.41 (0.87–2.23), *p* = 0.148
Lines of therapy at initial lymphoma diagnosis^a^ (*n* = 83)	0–1 vs. ≥2	0.38 (0.17–0.83), ***p* = 0.016**	0.45 (0.22–0.89), ***p* = 0.023**
Best response to first‐line SCNSL (*n* = 90)	CR/PR vs. SD/PD	0.24 (0.12–0.48), ***p* < 0.001**	0.08 (0.04–0.16), ***p* < 0.001**
HDT‐AHSCT for SCNSL	Yes vs. no	0.56 (0.40–0.78), ***p* < 0.001**	0.63 (0.49–0.82), ***p* < 0.001**
Remission prior HDT‐AHSCT^b^ (*n* = 30)	CR vs. PR	0.25 (0.06–0.96), ***p* = 0.044**	0.25 (0.09–0.65), ***p* = 0.005**
CAR T from second‐line SCNSL onwards^c^ (*n* = 56)	Yes vs. no	0.29 (0.09–0.97), ***p* = 0.045**	0.38 (0.16–0.90), ***p* = 0.029**

*Note*: Bold values indicates *p* ≤ 0.05.

Abbreviations: CR, complete remission; ECOG PS, Eastern Cooperative Oncology Group Performance Status; HDT‐AHSCT, high‐dose chemotherapy with autologous haematopoietic stem cell transplantation; OS, overall survival; PFS, progression‐free survival; PR, partial remission; SCNSL, secondary central nervous system lymphoma.

^a^ Only patients without SCNSL manifestation at initial diagnosis.

^b^ Only patients with HDT‐AHSCT.

^c^ Only patients with progression/relapse after first‐line SCNSL therapy and concomitant CNS involvement at progression/relapse.

HD‐MTX‐based regimens demonstrated improved OS compared with no HD‐MTX (Table S5). In pairwise analyses, both intensive HD‐MTX combination and reduced‐intensity HD‐MTX‐based therapy showed superior OS compared with no HD‐MTX or IT‐only approaches, while no significant difference was observed between intensive combinations and reduced‐intensity HD‐MTX‐based regimens (Table S6).

HDT‐AHSCT was administered to 31 patients (29.5%), mostly after first‐line therapy (*n* = 27, 25.7%). HDT‐AHSCT was associated with improved OS and PFS (median OS not reached vs. 10.8 months; median PFS 12.9 vs. 5.1 months), with significance in univariate (OS: HR 0.56, PFS: HR 0.63; both *p* < 0.001) and multivariate analyses (OS: HR 0.39, *p* = 0.013; PFS: HR 0.41, *p* = 0.002) (Tables 5 and 6; Figure 1C,D). Among HDT‐AHSCT recipients, CR before consolidation was associated with superior OS and PFS compared to PR (*p* = 0.029 and 0.003 respectively) (Table 4).

**TABLE 6:** Prognostic factors in SCNSL (multivariate Cox regression analysis).

Parameter	Versus	OS HR (95% CI), *p* (*n* = 94)	PFS HR (95% CI), *p* (*n* = 101)
Age in 10‐year units	—	1.20 (0.93–1.55), *p* = 0.169	0.93 (0.77–1.13), *p* = 0.465
ECOG PS at SCNSL	0–1 vs. ≥2	0.83 (0.47–1.47), *p* = 0.529	0.63 (0.39–1.00), ***p* = 0.050**
Ann Arbor at initial lymphoma diagnosis	1–2 vs. 3–4	0.84 (0.41–1.74), *p* = 0.644	0.84 (0.47–1.53), *p* = 0.577
ENM manifestation at initial lymphoma diagnosis	Yes vs. no	1.21 (0.51–2.89), *p* = 0.669	0.71 (0.36–1.39), *p* = 0.316
Testicle/kidney/adrenal gland involvement	Yes vs. no	0.84 (0.41–1.69), *p* = 0.616	0.88 (0.49–1.58), *p* = 0.668
HDT‐AHSCT	Yes vs. no	0.39 (0.18–0.82), ***p* = 0.013**	0.41 (0.23–0.71), ***p* = 0.002**

*Note*: Bold values indicates *p* ≤ 0.05.

Abbreviations: ECOG PS, Eastern Cooperative Oncology Group Performance Status; ENM, extra‐nodal manifestation; HDT‐AHSCT, high‐dose chemotherapy with autologous haematopoietic stem cell transplantation; OS, overall survival; PFS, progression‐free survival; SCNSL, secondary central nervous system lymphoma.

Among nine patients receiving CAR T therapy, eight achieved CR. Those treated from second‐line onwards (*n* = 7) had superior OS (31.0 vs. 11.9 months, *p* = 0.034) and PFS (9.5 vs. 4.3 months, *p* = 0.023) compared with other regimens (Table 4; Figure S2). Cox regression supported these associations (OS: HR 0.29, *p* = 0.045; PFS: HR 0.38, *p* = 0.029) (Table 5). Ibrutinib was used as salvage in six patients, with three achieving CR or PR (50.0%), but was not associated with a survival advantage (Table 4; Table S4).

## DISCUSSION

In this multicentre real‐world study, we provide a detailed characterization of consecutively treated patients with SCNSL, focusing on treatment feasibility, response dynamics and sequencing of therapeutic strategies. The cohort reflects routine clinical practice: nearly half of the 105 patients were >65 years, and 55.3% had ECOG PS ≥2 at SCNSL diagnosis, highlighting the frailty of this population. Thus, our dataset captures the limitations and clinical decision‐making challenges encountered in an unselected SCNSL population.

A clinically relevant observation is that outcome in SCNSL appears to be strongly determined by dynamic disease‐ and patient‐related factors, particularly PS, early response to CNS‐directed therapy and feasibility of intensive consolidation. ECOG ≥2 at SCNSL diagnosis was associated with significantly worse OS and PFS, whereas traditional prognostic indices such as the IPI and CNS‐IPI did not correlate with outcome. This highlights the relevance of dynamic clinical markers such as functional status and treatment response once CNS involvement has occurred.

Patients undergoing HDT‐AHSCT had longer PFS and OS than non‐transplanted patients, with 2‐year PFS of 44.3% vs. 13.7% and 2‐year OS of 66.6% vs. 34.9%. Among patients in CR before HDT‐AHSCT, 2‐year OS and PFS reached 82.5% and 46.2% respectively. The relatively low rate of consolidative HDT‐AHSCT likely reflects the consecutive real‐world nature of our cohort, including patients with a median age of 65 years and median ECOG of 2 at SCNSL diagnosis. Compared with cohorts reporting higher transplant rates and more favourable baseline fitness, this underscores that HDT‐AHSCT feasibility is strongly determined by PS, comorbidities, treatment response and early disease control. Our findings are consistent with the SCNSL‐R registry,^3^ the German multicentre study by Treiber et al.^2^ and the recent large cohort reported by Alderuccio et al.,^1^ supporting early response and consolidative HDT‐AHSCT as major prognostic factors. Together, these data support response‐adapted HDT‐AHSCT as a central curative‐intent strategy for eligible patients, while emphasizing that only a selected subset of real‐world patients can proceed to this approach.

First‐line HD‐MTX–based regimens were associated with superior outcomes than intrathecal‐only or non‐HD‐MTX approaches, consistent with current SCNSL management concepts supporting systemic CNS‐penetrating HD‐MTX–based therapy as the therapeutic backbone, rather than intrathecal alone.^4^, ^17^ In line with Alderuccio et al., we did not observe an additional benefit from combining HD‐MTX with intrathecal therapy compared with HD‐MTX‐based therapy alone.^1^ However, intensive CNS‐directed HD‐MTX combination regimens were not superior to reduced‐intensity HD‐MTX–based regimens such as R‐MP in our cohort, which may reflect the smaller sample size, greater comorbidity and treatment selection according to fitness.

Patients failing to respond to first‐line therapy had dismal outcomes, with median OS <3 months, underscoring the need for early response assessment and prompt transition to alternative strategies such as CAR T therapy or clinical trials. Detailed documentation of treatment failure patterns and subsequent therapy lines is a relevant strength of our study, as evidence after failure of MTX‐based CNS‐directed therapy remains limited.

Our data suggest a potential role of CAR T therapy in SCNSL. In our cohort, two patients received anti‐CD19 CAR T therapy during first‐line SCNSL treatment (and early relapse of systemic lymphoma) and seven in second or later treatment lines, all but one achieved CR, while one had SD. While outcomes appeared favourable compared with non‐transplant salvage approaches in univariate analysis, this finding is limited by the very small sample size and potential selection bias. CAR T therapy should therefore not be considered an alternative to consolidative HDT‐AHSCT in fit and responding patients but may represent a therapeutic option for selected patients with rr SCNSL after MTX‐based therapy, particularly when conventional options are limited or HDT‐AHSCT is not feasible. These findings align with emerging literature.^15^, ^18^, ^19^, ^20^ Epperla et al. reported an ORR of 68% (CR 57%) in 61 SCNSL patients, with median PFS of 3.3 months and OS of 7.6 months.^18^ While durability remains a concern, five out of seven later line CAR T‐treated patients in our cohort remained progression‐free beyond 1 year, suggesting durable remissions in selected patients.

Ibrutinib was administered to only six patients and was not associated with a survival advantage. Nevertheless, Bruton tyrosine kinase (BTK) inhibition remains biologically plausible in CNS lymphoma, with Grommes et al. reporting an ORR of 60% in 15 patients with rr SCNSL treated with ibrutinib monotherapy and a recent meta‐analysis reported a pooled ORR of 66.2% for SCNSL, albeit with substantial heterogeneity.^21^, ^22^

Several limitations should be acknowledged. The retrospective design, absence of centralized response adjudication and non‐randomized treatment selection may introduce bias. Subgroup analyses of later line treatments, including CAR T therapy and BTK inhibition, are limited by small patient numbers and should be interpreted descriptively. However, the multicentre design and consecutive real‐world cohort enhance the clinical relevance of the findings.

In summary, this study provides a contemporary multicentre real‐world analysis of consecutive patients with SCNSL, focusing on treatment feasibility, response‐adapted consolidation and salvage sequencing. Our findings reinforce the prognostic value of early treatment response and PS, support HDT‐AHSCT as the preferred consolidative approach in eligible patients and highlight CAR T therapy as a potential option for selected patients with rr SCNSL.

## AUTHOR CONTRIBUTIONS

**Farina Eigendorff:** Writing – original draft; methodology; conceptualization; formal analysis; writing – review and editing; investigation; resources; visualization. **Sophia Wollnitza:** Investigation; writing – review and editing; resources. **Ulf Schnetzke:** Conceptualization; investigation; writing – original draft; methodology; validation; visualization; writing – review and editing; formal analysis; supervision; resources. **Ellen Obstfelder:** Resources; writing – review and editing; investigation. **Evgenii Shumilov:** Resources; investigation; writing – review and editing. **Fabian Görke:** Writing – review and editing; methodology; writing – original draft; investigation; conceptualization; resources. **Vanja Zeremski:** Writing – review and editing; resources; investigation. **Georg Lenz:** Investigation; writing – review and editing; writing – original draft; methodology; resources; supervision. **Inken Hilgendorf:** Writing – review and editing; resources; investigation. **Vladan Vučinić:** Writing – review and editing; investigation; methodology; resources; writing – original draft. **Andreas Hochhaus:** Supervision; resources; investigation; writing – review and editing.

## FUNDING INFORMATION

No funding.

## CONFLICT OF INTEREST STATEMENT

FE received Honoraria and travel funds not related to this manuscript from Abbvie, Novartis, Roche; EO received Honoraria and travel funds not related to this manuscript from Kite, Beigene; IH received Honoraria and/or travel funds not related to this manuscript from Abbvie, Alexion, Amgen, Medac, Novartis Jazz Pharma, Sanofi Genzyme; VV received Research support not related to this manuscript from Amgen, Gilead/Kite and Honoraria not related to this manuscript from Gilead/Kite, Novartis, BMS Celgene, J&J, Sobi, Astrazeneca, Roche, Mallinckrodt, Elli Lilly and travel funds not related to this manuscript from Sanofi, Gilead/Kite, J&J, Astrazeneca; GL received research grants not related to this manuscript from Abbvie, AstraZeneca, Bayer, Gilead, MorphoSys and Sobi. GL received honoraria not related to this study from ADC Therapeutics, AbbVie, AstraZeneca, BeiGene, BMS, Genmab, Gilead, GSK, Hexal/Sandoz, Immagene/Flindr, Incyte, Lilly, Miltenyi, MorphoSys, MSD, Novartis, PentixaPharm, Pierre Fabre, F. Hoffmann‐La Roche Ltd. and Sobi.; AH received Research support not related to this manuscript from Novartis, BMS, Pfizer, Incyte, Terns, Enliven, Honoraria not related to this manuscript from Novartis, Incyte; US received Honoraria and travel funds not related to this manuscript from Abbvie, BeiOne, BMS, J&J, Kite, Novartis, SOBI.

## Supporting information


**Table S1.** Therapy for patients without SCNSL at initial diagnosis.
**Table S2.** Therapy for patients without SCNSL at initial diagnosis (second‐line therapy onwards).
**Table S3.** Therapy for SCNSL manifestation (third‐line therapy onwards).
**Table S4.** CAR T, ibrutinib.
**Table S5.** Impact of HD‐MTX and *i*th therapy on OS in first‐line SCNSL (log‐rank test).
**Table S6.** Pairwise comparison for impact of HD‐MTX and *i*th therapy on OS in first‐line SCNSL (post hoc test).
**Table S7.** Impact of SCNSL manifestation on PFS (log‐rank test).
**Table S8.** Pairwise comparison for impact of SCNSL manifestation on PFS (post hoc test).
**Figure S1.** Impact of SCNSL manifestation on PFS. Parenchymal versus meningeal versus combined.
**Figure S2.** (A) OS from relapse/progression after first‐line SCNSL therapy. CAR T versus no CAR T. (B) PFS from relapse/progression after first line SCNSL therapy. CAR T versus no CAR T.

## Data Availability

Data are available upon reasonable request from the patient records at theUniversity Hospital Jena, University Hospital Leipzig, University Hospital Magdeburg and University Hospital Muenster.
