# Supplementary material for: Response‐adapted consolidation and salvage strategies in secondary central nervous system lymphoma: Insights from a multicentre cohort
Source: Br J Haematol. 2026 Jun 25;209(2):524–33. doi: 10.1111/bjh.70633 (PMC13461960; doi:10.1111/bjh.70633)
Supplement: Supplementary file 1 [file BJH-209-524-s001.docx]

**Supplemental Table 1**

**Therapy for patients without SCNSL at initial diagnosis**

| **Patients without SCNSL manifestation at initial diagnosis, n (%)** | - | 83 (79.0) |
| --- | --- | --- |
| **1^st^ line therapy, n (%)** | R-CHO(E)P  R-CHO(E)P + Radiation  GMALL NHL protocol  Other* | 65 (78.3)  10 (12.0)  2 (2.4)  6 (7.2) |
| **Best response to 1^st^ line therapy, n (%)** | CR  PR  SD  PD  Not assessed | 53 (67.1)  16 (20.3)  2 (2.5)  7 (8.9)  1 (1.3) |
| **TTP1 (months), median (95% CI)** | - | 9.99 (8.81-11.16) |
| **Lymphoma manifestation after 1^st^ line therapy, n (%)** | **SCNS manifestation**  SCNSL only  SCNSL with concomitant systemic  manifestation  Systemic only  Not assessed | **71 (85.5)**  66 (79.5)  5 (6.0)  12 (14.5)  0 (0) |

*****Obinutuzumab-Bendamustine (n = 1), Obinutuzumab-CHOP (n = 1), Pola-R-CHP (n = 1), Rituximab mono + Radiation (n = 1), R-MTX (n = 1), alternative methods of healing (n = 1)

Abbreviations: *CHP* cyclophosphamide, doxorubicin, prednisolone; *CR* complete remission; *GMALL (-NHL)* German multicenter adult acute lymphoblastic leukemia (Non-Hodgkin lymphoma) protocol; *MTX* (high-dose) methotrexate; *PD* progressive disease; *Pola* Polatuzumab vedotin; *PR* partial remission; *R* rituximab; *R-CHO(E)P* rituximab, cyclophosphamide, doxorubicin, vincristine, (etoposide), prednisolone; *SD* stable disease; *TTP* time to progression.

**Supplemental Table 2**

**Therapy for patients without SCNSL at initial diagnosis (second line therapy onwards)**

| **2^nd^ line therapy, n (%)** | R-DHAP (+ HDT-ASCT)  (Pola)-R-Bendamustine  R-ICE (+ HDT-ASCT)  Other*  Total | 4 (33.3)  3 (25.0)  2 (16.7)  3 (25.0)  12 (100) |
| --- | --- | --- |
| **Best response to 2^nd^ line therapy, n (%)** | CR  PR  SD  PD | 7 (58.3)  3 (25.0)  1 (8.3)  1 (8.3) |
| **TTP2 (months), median (95% CI)** | - | 5.65 (0.01-11.67) |
| **Lymphoma manifestation after 2^nd^ line therapy, n (%)** | **SCNSL manifestation**  SCNSL only  SCNSL with concomitant systemic  manifestation  Systemic only | **6 (50.0)**  6 (50.0)  0 (0)  6 (50.0) |
| **3^rd^ line therapy, n (%)** | R-Pola-Benda (+ HDT-ASCT)  Radiation (+ HDT-ASCT)  Other** | 3 (50.0)  2 (33.3)  1 (16.7) |
| **Best response to 3^rd^ line therapy, n (%)** | CR  PR  SD  PD | 2 (33.3)  1 (16.7)  0 (0)  3 (50.0) |
| **TTP3 (months), median (95% CI)** | - | 2.33 (0.43-4.24) |
| **Lymphoma manifestation after 3^rd^ line therapy, n (%)** | **SCNSL manifestation**  SCNSL only  SCNSL with concomitant systemic  manifestation  Systemic only | **4 (66.7)**  3 (50.0)  1 (16.7)  2 (33.3) |
| **4^th^ line therapy, n (%)** | CAR T (+ Radiation)  other | 2 (100)  0 (0) |
| **Best response to 4^th^ line therapy, n (%)** | CR  PR  SD  PD | 2 (100)  0 (0)  0 (0)  0 (0) |
| **TTP4 (months), median (95% CI)** | - | 5.68 (n.a.) |
| **Lymphoma manifestation after 4^th^ line therapy, n (%)** | **SCNSL manifestation**  SCNSL only  SCNSL with concomitant systemic  manifestation  Systemic only | **2 (100)**  2 (100)  0 (0)  0 (0) |

*Pola-R-CHP (n = 1), Tafasitamab/Lenalidomide (n = 1), Surgery (Vitrectomy) (n = 1); ******Surgery (Vitrectomy) (n = 1)

Abbreviations: *CAR T* chimeric antigen receptor T-cell therapy; *CHP* cyclophosphamide, doxorubicin, prednisolone; *HDT-ASCT* high-dose chemotherapy with autologous stem cell transplantation; *PD* progressive disease; *Pola* Polatuzumab vedotin; *R-DHAP* rituximab, dexamethasone, high-dose cytarabine, cisplatin; *R-ICE* rituximab, ifosfamide, carboplatin, etoposide.

**Supplemental Table 3**

**Therapy for SCNSL manifestation (third line therapy onwards)**

| **3^rd^ line therapy, n (%)** | BSC  Radiation only  Ibrutinib  Other* | 8 (38.1)  6 (28.6)  2 (9.5)  5 (23.8) |
| --- | --- | --- |
| **Additional therapy to 3^rd^ line therapy, n (%)** | Add. Intrathecal therapy  Add. HDT-ASCT  Add. Radiation  Total | 1 (4.8)  1 (4.8)  1 (4.8)  3 (14.3) |
| **Best response to 3^rd^ line therapy, n (%)** | CR  PR  SD  PD  Not assessed | 3 (14.3)  5 (23.8)  0 (0)  3 (14.3)  10 (47.6) |
| **TTP3 (months), median (95% CI)** | - | 2.79 (0.97-4.62) |
| **Lymphoma manifestation after 3^rd^ line therapy, n (%)** | **SCNS manifestation**  SCNSL only  SCNSL with concomitant systemic  manifestation  Systemic only | **5 (55.6)**  3 (33.3)  2 (22.3)  4 (40.0) |
| **4^th^ line therapy, n (%)** | BSC  Radiation  Other** | 3 (60.0)  1 (20.0)  1 (20.0) |
| **Best response to 4^th^ line therapy, n (%)** | CR  PR  SD  PD  Not assessed | 1 (20.0)  0 (0)  0 (0)  1 (20.0)  3 (60.0) |
| **TTP4 (months), median (95% CI)** | - | 2.14 (n.a.) |
| **Lymphoma manifestation after 4^th^ line therapy, n (%)** | **SCNS manifestation**  SCNSL only  SCNSL with concomitant systemic  manifestation  Systemic only | **3 (100)**  1 (33.3)  2 (66.6)  0 (0) |

*Burkitt protocol (n = 1), Pembrolizumab (n = 1), R-GDP (n = 1), unknown (n = 2); **R-Pola-Bendamustine + allogenic PBSCT (n = 1)

Abbreviations: *BSC* best supportive care; *n.a.* not available; *R-GDP* rituximab, gemcitabine, dexamethasone, cisplatin*;* *PBSCT* peripheral blood stem cell transplantation.

**Supplemental Table 4 CAR T, Ibrutinib**

| **CAR T for SCNSL, n (%)** | Yes  No | 9 (8.6)  96 (91.4) |
| --- | --- | --- |
| **CAR T product, n (%)** | Axi-cel  Tisa-cel  Liso-cel | 5 (55.6)  3 (33.3)  1 (11.1) |
| **Bridging regime prior CAR T, n (%)** | Immunochemotherapy  Radiation  No bridging | 6 (66.7)  1 (11.1)  2 (22.2) |
| **CNS manifestation at time of CAR T indication, n (%)** | SCNSL only  SCNSL with concomitant systemic manifestation | 4 (44.4)  5 (55.6) |
| **Remission prior CAR T, n (%)** | CR  PR  SD  PD  Not assessed | 7 (77.8)  2 (22.2)  0 (0)  0 (0)  0 (0) |
| **Best response to CAR T, n (%)** | CR  PR  SD  PD  Not assessed | 8 (88.9)  0 (0)  1 (11.1)  0 (0)  0 (0) |
| **Ibrutinib for SCNSL, n (%)** | Yes  No | 6 (5.7)  99 (94.3) |
| **Remission prior Ibrutinib, n (%)** | CR  PR  SD  PD  Not assessed | 0 (0)  3 (50.0)  0 (0)  2 (33.3)  1 (16.7) |
| **Best response to Ibrutinib, n (%)** | CR  PR  SD  PD  Not assessed | 1 (16.7)  2 (33.3)  2 (33.3)  0 (0)  1 (16.7) |

Abbreviations: *Axi-cel* Axicabtagene ciloleucel; *Liso-cel* Lisocabtagene maraleucel; *Tisa-cel* Tisagenlecleucel.

**Supplemental Table 5**

**Impact of HD-MTX and i.th. therapy on OS in 1^st^ line SCNSL (log-rank test)**

| **Group** | **description** | n (%) |
| --- | --- | --- |
| 1 | No HD-MTX | 11 (11.1) |
| 2 | Intensive HD-MTX CNS-directed combination therapy* | 42 (42.4) |
| 3 | Reduced-intensity HD-MTX based therapy** | 27 (27.3) |
| 4 | HD-MTX anytime AND i.th. therapy | 13 (13.1) |
| 5 | No HD-MTX, i.th. therapy only | 6 (6.1) |

Log rank test for OS: **p < 0.001**

*MATRIX, HD-MTX-based combination therapy with HD-AraC, Ifosfamide and/or Thiotepa

**RM(P), HD-MTX-based combination therapy with non-CNS-directed backbone (e.g. R-CHOP)

**Supplemental Table 6**

**Pairwise comparison for impact of HD-MTX and i.th. therapy on OS in 1^st^ line SCNSL (post-hoc test)**

| **Pair** | **n (%)** | **p-value** |
| --- | --- | --- |
| 1 vs. 2 | 53 (53.5) | **< 0.001** |
| 1 vs. 3 | 38 (38.4) | **< 0.001** |
| 1 vs. 4 | 24 (24.2) | **< 0.001** |
| 1 vs. 5 | 17 (17.2) | 0.486 |
| 2 vs. 3 | 69 (69.7) | 0.563 |
| 2 vs. 4 | 55 (55.6) | 0.291 |
| 2 vs. 5 | 48 (48.5) | **0.017** |
| 3 vs. 4 | 40 (40.4) | 0.613 |
| 3 vs. 5 | 33 (33.3) | **0.046** |
| 4 vs. 5 | 19 (19.2) | 0.098 |

**Supplemental Table 7**

**Impact of SCNSL manifestation on PFS (log-rank test)**

| **Group** | **description** | n (%) |
| --- | --- | --- |
| 1 | parenchymal | 60 (57.1) |
| 2 | meningeal | 32 (30.5) |
| 3 | ocular | 1 (1.0) |
| 4 | spinal | 1 (1.0) |
| 5 | combined | 11 (10.4) |

Log rank test for PFS: **p = 0.036**

**Supplemental Table 8**

**Pairwise comparison for impact of SCNSL manifestation on PFS (post-hoc test)**

| **Pair** | **n (%)** | **p-value** |
| --- | --- | --- |
| 1 vs. 2 | 92 (53.5) | **0.019** |
| 1 vs. 5 | 71 (38.4) | **0.019** |
| 2 vs. 5 | 43 (24.2) | 0.052 |

Group 3 and 4 were excluded from post-hoc analysis due to the small sample size (n = 1, respectively).

**Supplemental Figure 1**

**Impact of SCNSL manifestation on PFS.** Parenchymal versus meningeal versus combined

**Supplemental Figure 2**

**Supplemental Figure 2A OS from relapse/progression after 1^st^ line SCNSL therapy.** CAR T versus no CAR T

Abbreviations: *NE* not estimable

**Supplemental Figure 2B PFS from relapse/progression after 1^st^ line SCNSL therapy.** CAR T versus no CAR T
